# Magnetic resonance imaging and ultrasound elastography in the context of preclinical pharmacological research: significance for the 3R principles

**DOI:** 10.3389/fphar.2023.1177421

**Published:** 2023-06-28

**Authors:** Michael Obrecht, Stefan Zurbruegg, Nathalie Accart, Christian Lambert, Arno Doelemeyer, Birgit Ledermann, Nicolau Beckmann

**Affiliations:** ^1^ Diseases of Aging and Regenerative Medicines, Novartis Institutes for BioMedical Research, Basel, Switzerland; ^2^ Neurosciences Department, Novartis Institutes for BioMedical Research, Basel, Switzerland; ^3^ 3Rs Leader, Novartis Institutes for BioMedical Research, Basel, Switzerland

**Keywords:** magnetic resonance imaging, ultrasound elastography, small rodent, pharmacology, translational research, preclinical research, *in vivo* imaging, 3R principles

## Abstract

The 3Rs principles—reduction, refinement, replacement—are at the core of preclinical research within drug discovery, which still relies to a great extent on the availability of models of disease in animals. Minimizing their distress, reducing their number as well as searching for means to replace them in experimental studies are constant objectives in this area. Due to its non-invasive character *in vivo* imaging supports these efforts by enabling repeated longitudinal assessments in each animal which serves as its own control, thereby enabling to reduce considerably the animal utilization in the experiments. The repetitive monitoring of pathology progression and the effects of therapy becomes feasible by assessment of quantitative biomarkers. Moreover, imaging has translational prospects by facilitating the comparison of studies performed in small rodents and humans. Also, learnings from the clinic may be potentially back-translated to preclinical settings and therefore contribute to refining animal investigations. By concentrating on activities around the application of magnetic resonance imaging (MRI) and ultrasound elastography to small rodent models of disease, we aim to illustrate how *in vivo* imaging contributes primarily to reduction and refinement in the context of pharmacological research.

## Introduction

The 3R concept (replacement, reduction, and refinement) concerning the humane treatment of experimental animals was introduced in 1959 by Russell and Burch in the book *The Principles of Humane Experimental Technique* (Russell and Burch, 1959). The development and use of methods that improve the animal welfare by minimizing eventual stress, discomfort and/or pain during experimentation, as well as by reducing the number of animals are central for achieving ethical, scientific and even economic benefits. Moreover, despite ongoing efforts to substitute animal experiments by *in vitro* or *in silico* methods, significant challenges arise in the study of complex regulatory processes of the cardiovascular, metabolic, respiratory or nervous systems, for instance, or in the investigation of pathology, especially when the disease mechanisms are poorly understood. Thus, animal experimentation remains central to examine disease as well as in the context of drug discovery. Traditional experiments rely heavily on invasive techniques necessitating to sacrifice animals during the course of a study in order to perform, e.g., histopathological analyses. Often such invasive approaches are limited when it comes to identify crucial steps about disease progression or compound effects.

Non-invasive *in vivo* imaging provides potential to quantify with minimal distress anatomical, functional, metabolic or molecular alterations within the animal’s body. Imaging allows monitoring temporally and spatially animal models of diseases and the response to therapy (Rudin and Weissleder, 2003; Beckmann, 2006; Ripoll et al., 2008; Beckmann and Garrido, 2013). Through examples primarily from our own experience in adopting imaging in the context of pharmacological studies in small rodent disease models, we aim at illustrating the win-win situation between animal welfare and the relevance of data obtained from animal studies. Non-invasive imaging enables to reduce significantly the number of animals used for experimentation. Repeated measurements allow each animal to serve as its own control, thereby benefitting statistical analyses and resulting in an estimated reduction of more than 80%, depending on the application and the study protocol. For instance, in a rat model of prolactinoma in which pituitary hyperplasia was induced by chronic stimulation with estradiol, a large variability in pituitary volume was observed, which translated into a coefficient of variation of 80% (Rudin et al., 1988). Thus, in order to detect a statistically significant (*p* = 0.05) 50% volume decrease upon treatment, group sizes of at least 35 animals would be required if weighting the pituitary would be the endpoint. However, monitoring the pituitary volume by imaging resulted in a coefficient of variation of 12% and a sample size of *n* = 4 rats were sufficient to reach the same level of statistical significance (Rudin et al., 1988). By repeated examinations of individual mice, animal numbers could be reduced from 96 to 16 mice in a stroke model by incorporating various imaging modalities (Barca et al., 2021). Also, 12 rats were sufficient to longitudinally quantify lung inflammation in an ovalbumin model and to detect compound effects (Tigani et al., 2003). When adopting the traditional terminal method of bloncho-alveolar lavage (BAL) fluid analysis, 96 rats would have been necessary. Moreover, the early resolution of edematous signals quantified by imaging upon anti-inflammatory drugs did not involve general suppression of the inflammatory response monitored by traditional BAL fluid analysis (Tigani et al., 2003). In other words, the effect of the compounds on the influx of inflammatory cells in the airways as quantified in BAL was delayed with respect to the effect at the tissue level, as revealed by MRI.

A distinct advantage of imaging is the ability to go back and reanalyze images. Thus, when there are new biological questions/insights there is the option to avoid re-running animal experiments by first re-probing old images. Moreover, there is the possibility to generate information not accessible to *ex vivo* or *post-mortem* approaches, especially regarding functional assessments. This experimental refinement is enabled by the fact that imaging analyzes the organ *in situ* in the intact organism. All these features bear great relevance in the framework of *in vivo* pharmacology, in particular when addressing therapeutic effects of compounds. Indeed, testing compounds upon established pathology rather than under preventative conditions is of paramount importance. In the case of preclinical experimentation with disease models, it can be expected that every animal reacts differently to a pathological stimulus. Imaging provides the opportunity to non-invasively quantify the pathology just before initiation of treatment. This has an important practical implication as animals can then be randomized into the different treatment groups to have equivalent mean pathology distribution just before initiation of compound or vehicle dosing. Alternatively, the pathology status pre- and post-administration of compound or vehicle can be easily compared in each animal. *Post-mortem* analyses do not allow such direct comparison.

Various imaging techniques including “micro” X-ray computed tomography (micro-CT), position emission tomography (PET), single photon emission computed tomography (SPECT), bioluminescence, fluorescence imaging, magnetic resonance imaging (MRI) and ultrasound, have been used to study biology in small rodent models of diseases (Rudin and Weissleder, 2003; Beckmann et al., 2007; Cunha et al., 2014; Tremoleda and Sosabowski, 2015; Lauber et al., 2017). Within pharmacological research, optical imaging (bioluminescence, near infrared fluorescence imaging) and nuclear medicine techniques (PET, SPECT) are used to address questions related to target engagement, compound distribution and pharmacokinetics (Ripoll et al., 2008; Gomes et al., 2011; Fernandes et al., 2012; Razansky et al., 2012; Jang, 2013; Sharma, 2017). Moreover, the development of radiotheranostic probes enable diagnosis and treatment to be performed with the same agent, particularly in the cancer field (Colombo et al., 2017). Micro-CT, MRI and ultrasound on the other hand provide information on pharmacodynamic effects of compounds on structure and function. Here, attention is going to be limited to applications of MRI or ultrasound shear wave elastography (SWE) (Taljanovic et al., 2017) to pharmacological research in several disease areas incorporating the use of small rodent disease models to highlight the value of imaging in the context of animal welfare. In addition to reduction, these translational techniques enable to apply learnings from the clinics to refine and improve the animal models. Indeed, we aim to illustrate the importance of keeping the clinical picture in mind when performing preclinical pharmacological assessments in small rodents.

### *In vivo* imaging: A few considerations

In most of the *in vivo* imaging applications, animals are anesthetized during the acquisitions. Potential effects of anesthesia need to be attentively considered, as they may not only impact functional acquisitions but also interfere with pharmacological studies. For the majority of the examples discussed in this article, animals were anesthetized with isoflurane, the most widely adopted anaesthetic for laboratory animal imaging (Tremoleda et al., 2012), in air or O_2_ administered via a nose cone. Healthy small rodents easily recover from gas anesthesia in a few minutes, but additional burden may occur in diseased animals. It is important to carefully conceive the studies by considering the number of times an animal is anesthetized and the minimum interval between sequential imaging sessions. As guidance, imaging sessions with a duration under 30 min including positioning of the animals and a minimum interval of 3 h between sequential anesthesias are recommended. No special animal preparation is necessary for MRI or SWE examinations, a minor but important contribution towards refinement. Intravenous administration of a contrast agent is required only occasionally, for instance to verify the leakiness of the blood-brain-barrier. In this case, contrast material approved for clinical use is utilized.

In the past few years, efforts were pursued to reduce the acquisition times by employing image denoising strategies based on filtering or convoluted neural networks. For instance, denoising has been demonstrated to improve small rodent MRI of the heart (Delattre et al., 2012; Tricot et al., 2017), the kidney (de Senneville et al., 2020; Starke et al., 2021) and the central nervous system (Wells et al., 2010; Kim et al., 2016; Wang et al., 2019) as well as spectroscopic analyses (Simões et al., 2022). Even functional connectivity assessments may profit from denoising, as shown in a rat model of sporadic Alzheimer’s disease (Diao et al., 2021). Alternative acquisition protocols have been devised to reduce the acquisition times, allowing, e.g., displacements to be detected with temporal resolutions down to 5.5 ms which may benefit cardiac MRI (Lee et al., 2021) and to accelerate the acquisition of anatomical brain images (Spencer Noakes et al., 2017) as well as of diffusion (Lu L. et al., 2012a) or perfusion data (Gao et al., 2014). Another means to reduce the total acquisition time is to combine acquisitions, for instance for assessing two relaxation times simultaneously (Thomas et al., 2002; Liu et al., 2010). More details can be found in specialized reviews (Deshmane et al., 2012; Setsompop et al., 2016; Curtis and Cheng, 2022). Moreover, advancements in data reconstruction, particularly on weighted Compressive Sensing (Kumar et al., 2021) and on model-based or data-driven deep learning tools (Johnson et al., 2020; Wang et al., 2021; Pal and Rathi, 2022; Potočnik et al., 2023) further reduce aliasing artifact problems and improve signal-to-noise, therefore impacting acquisition times. Of note, many of these developments have been or are being realized on clinical systems. Their adaptation, validation and stability on small animal scanners still needs to be properly addressed.

Prior to being useful for pharmacological studies, *in vivo* imaging readouts need to be carefully validated against standard measures, in general obtained through terminal examinations. Well validated readouts are more relevant, ultimately impacting animal welfare. When histology serves as reference, the importance of having quantitative parameters based on image analysis rather than relying on qualitative scores needs to be stressed. Tissue histopathology slides stored in digital image format are assessable to computerized image analysis tools and machine learning techniques (Komura and Ishikawa, 2018; Hoefling et al., 2021).

Advances in genomic, transcriptomic, proteomic and metabolomic sciences are enabling research into complex diseases. This development is paving the way for the atomic resolution of diseases. Important insights into the genetic basis of human disease are being brought by genome-wide association analyses, while systems biology approaches enhance the understanding of disease mechanisms by addressing networks, pathways and targets (Yan et al., 2018). Combining imaging providing detailed anatomical and functional information of tissues and organs of the body with -omics approaches provides potential for improved diagnostics and better understanding disease progression, as shown recently in the context of oncology (Zhu et al., 2015; Borgheresi et al., 2022; Fathi Kazerooni et al., 2022), multiple sclerosis (Herman et al., 2018) as well as mild cognitive impairment and Alzheimer’s disease (Saykin et al., 2015) to name a few. Pursuing integration of imaging and -omics techniques in small rodents (Chakraborty et al., 2017) opens the door for an improved characterization of models of disease without the necessity of increasing animal usage.

Complex questions can be better addressed through open collaboration between groups at several institutions rather than individually. Exchange of information and experience can improve output quality through, e.g., understanding the factors leading to successful acquisitions, enhanced protocols, and/or data analysis. Such collective efforts may on the long run potentiate future data collection, improve standards, comparability and reproducibility, and ultimately contribute to a reduction of animal use by a diminution of discards. Examples are efforts around standardization of resting state functional MRI (rs-fMRI) in mice (Grandjean et al., 2020) and rats (Grandjean et al., 2023) involving multiple groups around the world mentioned below.

In the next sections, applications of MRI or SWE to quantify pathology in the musculoskeletal system, brain, lung, and liver in the context of small rodent disease models for pharmacological research are discussed keeping the animal welfare in mind. Also safety analyses using imaging are presented.

### Musculoskeletal system

#### Osteoarthritis

Osteoarthritis (OA) is a main cause of disability in older adults. Pain, loss of function and decreased quality of life are among the consequences of this long-term disease frequently affecting knee joints (Hunter et al., 2014). Traditionally OA was considered as a “wear and tear” condition, with articular cartilage damage, inflammation, stiffness, swelling, and loss of mobility resulting from a chronic overload and impaired biomechanics of the joint. Now it is known that OA involves a much more complex process orchestrated by inflammatory and metabolic factors in which the entire joint is affected, most notably the cartilage but also the synovium, joint ligaments, menisci and subchondral bone (Loeser et al., 2012; Martel-Pelletier et al., 2016).

In the absence of disease-modifying compounds, symptomatic treatments and, ultimately, joint replacement are currently the only therapeutic options available for knee OA. Small rodent models play an important role when testing new therapies (Kuyinu et al., 2016). Injury induced in rats by surgery leads to fast cartilage degenerative changes involving chondrocyte/proteoglycan loss and fibrillation as well as osteophyte formation. MRI has been applied in conjunction with such models to evaluate treatments (Huang et al., 2010; Huang et al., 2021; Gu et al., 2015; Mohan et al., 2016). The basis for the use of MRI is the quantification of degenerative cartilage compositional changes by T_2_ mapping, which is sensitive to abnormalities of the cartilage extracellular matrix including collagen fiber orientation (Joseph et al., 2018). Early phases of cartilage degeneration occurring prior to macroscopic cartilage defects and thinning are detectable, increases in T_2_ being associated with cartilage abnormalities (Pan et al., 2011).

**FIGURE 1:**
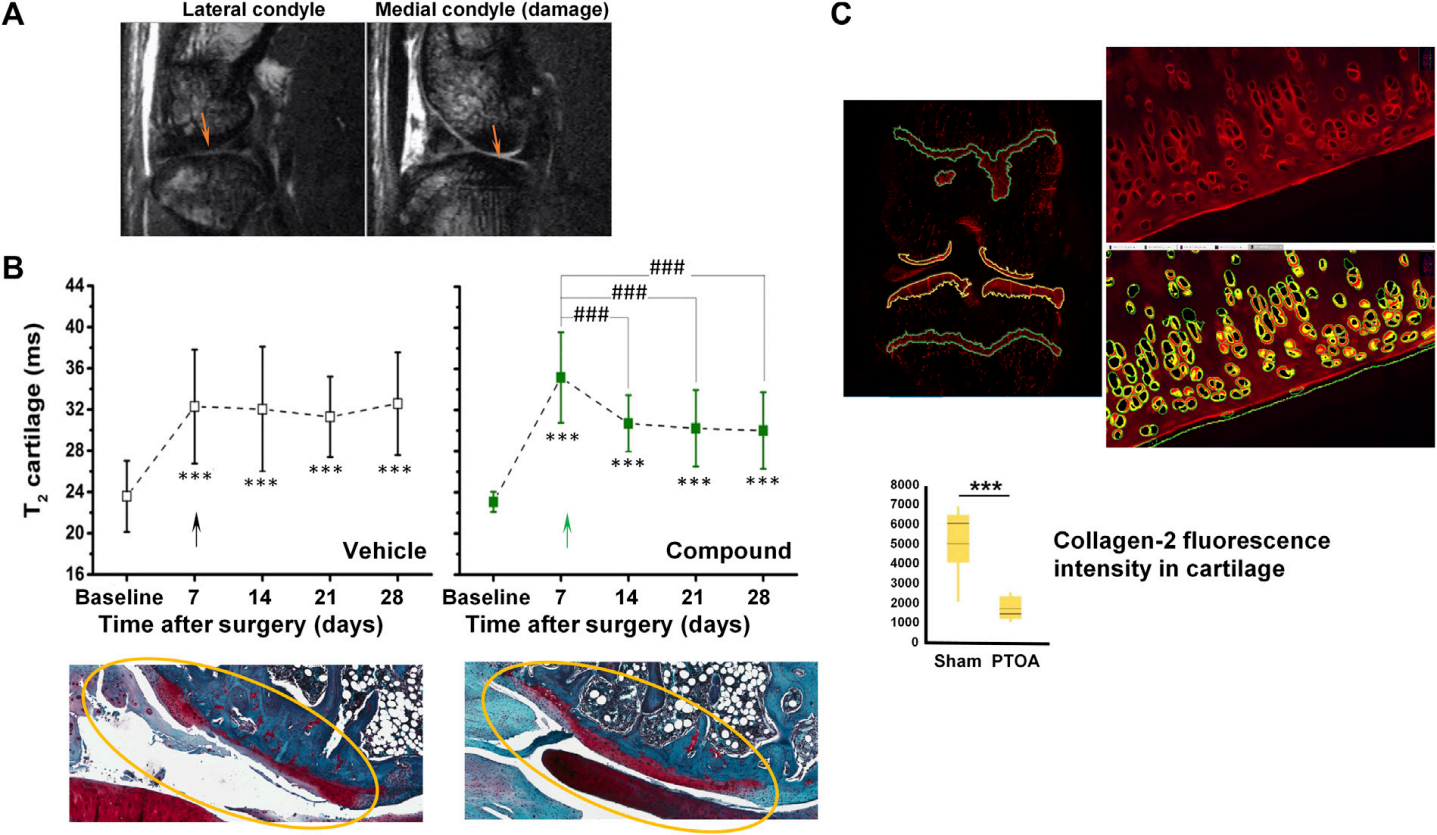
MRI in a post-traumatic OA (PTOA) rat surgical model of cartilage injury. **(A)** Spin-echo images acquired at 7 T from the same joint, 7 days after surgery. Damaged cartilage in the medial condyle displayed higher signal intensity than in the lateral condyle (arrows). **(B)** Higher signal intensity translated into increased T_2_ relaxation times in damaged cartilage. A reduction of T_2_ was observed upon intra-articular administration 1 week after injury onset of a compound aiming to promote cartilage regeneration. Representative safranin-O stained histological images confirmed the beneficial effect of the compound. **(C)** Quantification of fluorescence applying machine learning tools to immunohistochemistry for collagen-2. See Accart et al. (2022) for details on image acquisition and quantification. ^©^The Authors 2022.

Figure 1A shows images acquired in 8.5 min at 7 T from a rat knee joint after meniscal tear and medial collateral ligament transection. Cartilage appears brighter in the injured condyle, consistent with increased relaxation time T_2_ upon cartilage damage as illustrated in Figure 1B. Of note, the T_2_ of healthy cartilage of ∼20 ms in rats is consistent with values reported for hyaline cartilage in humans at the same field strength, ranging between 19 and 24 ms (Juras et al., 2016). Treatment with a compound administered intra-articularly at week 1 after surgery, aiming to promote cartilage regeneration, led to significant decrease of T_2_, suggesting beneficial effects on cartilage confirmed by histology at the end of the study (Figure 1B). Quantitative histological analysis supported the validation of T_2_ as non-invasive marker of cartilage damage in this surgical model (Figure 1C).

Modulation of cartilage T_2_ upon experimental treatment has also been reported in the clinic for knee OA patients. In a phase I–II trial T_2_ reductions upon intra-articular administration of *ex vivo* expanded autologous mesenchymal stromal cells indicated cartilage regeneration (Soler et al., 2016). Three recent studies demonstrated T_2_ reductions after implanting a biomaterial scaffold seeded with chondrocytes (Li et al., 2021; Xu et al., 2021; Janacova et al., 2022). In two of the studies early efficacy suggesting cartilage repair translated into cartilage T_2_ reductions within 3–6 months of implantation, accompanied by improvements in functionality and pain levels (Li et al., 2021; Xu et al., 2021). Texture analysis of T_2_ quantitative maps has also been introduced to study cartilage repair (Janacova et al., 2022).

The investigation by MRI of the knee joint as an organ with several tissues in focus was pursued in a rat study examining the effects of repeated intra-articular injections of monosodium urate (MSU) crystals with inflammasome priming by lipopolysaccharide (LPS) with the aim to simulate recurrent bouts of gout (Accart et al., 2022). Gout is a common form of arthritis, involving recurrent episodes of painful acute inflammatory flares in response to MSU crystals depositing mainly in peripheral joints (Kuo et al., 2015). The longstanding accumulation of MSU crystals can then elicit damage in the joints. Gout and osteoarthritis (OA) often occur in conjunction. Nonetheless, despite the positive correlation between the uric acid amount in the synovial fluid and OA (Denoble et al., 2011), currently it remains unknown whether gout and OA are pathologically linked (Jarraya et al., 2022). Repeated intra-articular administration of MSU/LPS to rats resulted in joint swelling, synovial membrane thickening, fibrosis of the infrapatellar fat pad, tidemark breaching, and incursion of inflammatory cells to cartilage (Accart et al., 2022). In comparison to saline administration, animals receiving MSU/LPS displayed higher pain sensitivity to van Frey filament stimulation of the hind-paws. In the joints of rats challenged with MSU/LPS, MRI showed an increase of synovial fluid volume related to inflammation, changes in the infrapatellar fat pad consistent with a progressive decrease of fat volume and fibrosis development, and a progressively increased T_2_ in femoral cartilage, in agreement with a reduced proteoglycan content in the same area. MRI displayed as well cyst formation in the tibia, femur remodeling, and T_2_ reductions in extensor muscles, the latter consistent with fibrosis generation in this tissue (Accart et al., 2022).

From the 3R’s perspective, benefits of including MRI in longitudinal preclinical OA studies are many fold: 1) A reduction by at least 80% in animal usage is estimated; 2) multiple tissues of the knee joint can be analyzed in an acquisition time of 8.5 min; 3) as injury may be heterogeneous, especially in the surgical models, randomizing animals into groups just before initiation of treatment using, e.g., cartilage T_2_ as measure contributes to reduce variability in pharmacological studies; and 4) the demonstration that cartilage T_2_ can be modulated by therapy both in preclinical animal studies and in OA patients strengthens the translational potential of the readout. Also interesting is the positive correlation between the relaxation time T_2_ in the infrapatellar fat pad and the latency time in the von Frey stimulation as a surrogate of pain sensitivity for the repeated intra-articular injections of MSU/LPS in rats (Accart et al., 2022). The infrapatellar fat pad is richly innervated (Lehner et al., 2008) and may be a source of pain in OA (Belluzzi et al., 2019). Trauma can lead to inflammation and eventually fibrotic lesions, both being sources of pain at the level of the infrapatellar fat pad (Eymard and Chevalier, 2016). Despite the positive correlation mentioned before, additional research is necessary to draw conclusions about the value of infrapatellar fat pad T_2_ as a surrogate marker of increased pain sensitivity in the MSU/LPS-induced knee joint injury or in other OA models.

#### Tendon injury

Tendons composed primarily of highly structure collagen fibers connect and transmit forces from the muscle to bone (Docheva et al., 2015). The stiffness of the tendon is critical for such an interaction. Achilles tendon rupture is often accompanied by substantial morbidity, mobility impairment, and increased absence from work. The recovery process of tendon stiffness following an injury is poorly understood, and the decision to return to full weightbearing for normalizing daily activities and practicing sports is solely based on clinical features.

Shear wave elastography (SWE) and wearable insoles evaluating tendon stiffness and foot plantar pressure, respectively, were examined with the aim to verify the feasibility of deriving objective quantitative measures after Achilles tendon rupture to ultimately facilitate decision making (Laurent et al., 2020). Over the 12-week duration of the study, the tendon stiffness in contralateral healthy tendons remained stable. In contrast, at week-2 post-injury the stiffness of the injured tendon was significantly decreased, most prominently in regions close to the rupture. Near complete stiffness recovery was detected at week 8 in distal regions to the rupture. However, at week 12 the stiffness in the proximal region of the injured Achilles was still significantly below that of the contralateral tendon. Despite the fact that the injured leg reached full weight-bearing capacity at week 12 after the injury, the plantar pressure distribution during walking showed slight sub-optimal function of the affected foot at this time point. Significant correlations between tendon shear wave velocity, insole variables and distinct activities indicated the clinical relevance of SWE and foot plantar pressure assessments (Laurent et al., 2020).

With translational research in mind, a validation preclinical study involving a rat model of tenotomy compared *in vivo* tendon stiffness measurements by SWE with *ex vivo* assessments of the Young’s modulus. A strong correlation (*R* ^2^ = 0.87, p = 5 × 10^−12^) was found between the tendon shear wave velocity measured *in vivo* and the *ex vivo* values of the Young’s modulus determined using biomechanics assays (Laurent et al., 2020). The 3R value of this experiment resides in a refinement, attesting to the adequacy of SWE for the quantification of tendon stiffness.

Ultrasound imaging and MRI were also integral part of a recent study describing enhanced tendon healing by a tough hydrogel with an adhesive side and a high drug-loading capacity (Freedman et al., 2022). Tissue adherent respectively gliding properties on opposing surfaces were displayed by this so-called Janus tough adhesive (JTA), which enabled drug delivery to the tendon tissue. A dual interpenetrating hydrogel network combining an alginate hydrogel and a highly elastic covalently cross-linked acrylamide hydrogel yielded the high JTA mechanical toughness (Sun et al., 2012). Tissue adhesion resulted from the unilateral coupling of the dissipative alginate acrylamide hydrogel to the amine rich bridging polymer chitosan (Li et al., 2017). The ability of the JTA to simultaneously support mechanical tissue integrity and spatially as well as temporally control drug delivery was demonstrated in rat models of tendon injury (Freedman et al., 2022).

#### Peripheral nerve injury

Peripheral nerve injury constitutes a major clinical and public health problem, often resulting in significant functional impairment, permanent disability and/or chronic pain (Modrak et al., 2020). Despite existing in varying severities, trauma to connective tissue, myelin, and axons is present in most forms of nerve injury. Microsurgery is currently the treatment of choice, but functional recovery following nerve repair is often unsatisfactory. Thus, new therapeutic strategies are necessary to increase functional recovery following injury.

**FIGURE 2:**
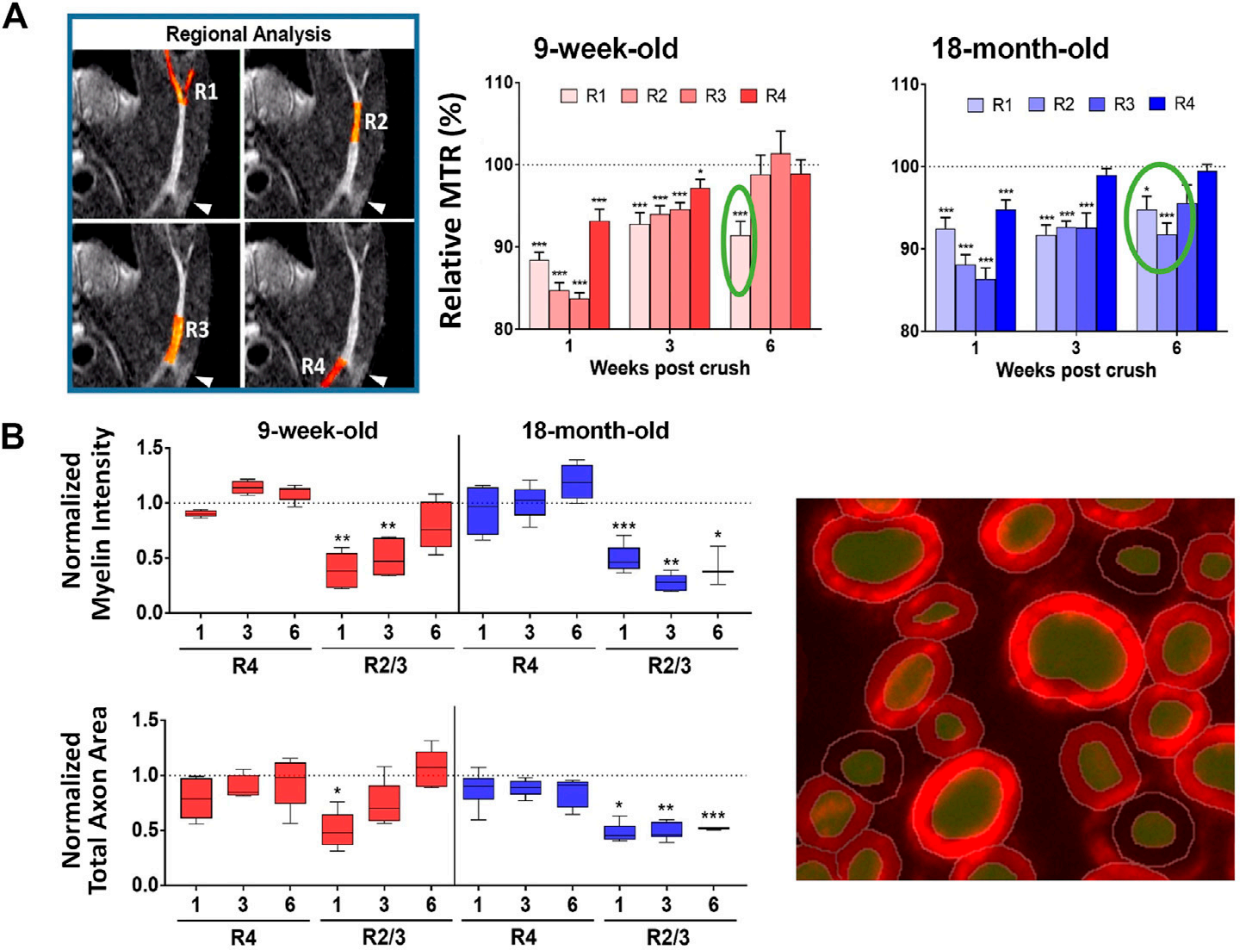
Sciatic nerve crush in mice. **(A)** Significantly lower MTR at different regions of the sciatic nerve were determined 1 week post-injury suggesting demyelination. At week 6, MTR was still below baseline after the nerve bifurcation in young mice, while for old mice the region displaying MTR below baseline was larger. **(B)** Quantitative histological analyses using machine learning tools of myelin basic protein fluorescence signal confirmed lower myelin content in the sciatic nerve as a function of time after the crush. See Giorgetti et al. (2019) for details. ^©^The Authors 2019.

Nerve crush is commonly used as experimental model to study recovery to peripheral nerve injury in small rodents (Bridge et al., 1994). Magnetization transfer ratio (MTR) reflecting myelin content in tissue (van der Weijden et al., 2021) as assessed non-invasively by MRI was adopted when investigating a model of nerve injury in which the sciatic nerve of mice was crushed using a forceps applied gently for 15 s. At baseline, before the injury onset, MTR in the sciatic nerve was significantly smaller in old compared to young animals. In healthy humans, MTR of lower extremity nerves has been found as well to decrease with age (Kollmer et al., 2018). Nerve demyelination elicited by the crush was reflected by reduced MTR in the first week after the crush in several areas along the nerve, with a partial recovery at later time points (Giorgetti et al., 2019) (Figure 2A). For young mice, at week 6 MTR in the nerve region after the bifurcation was still significantly below baseline. For old mice, the region displaying MTR below baseline was larger. Histology confirmed a larger area of demyelinated nerve in old compared to young mice at week 6 after the crush (Figure 2B). In other words, *in vivo* MRI and histology revealed an age-related impairment of nerve regeneration after crush (Giorgetti et al., 2019). Of note, electrophysiological recordings for young mice were back to baseline values at week 6. Also, muscle atrophy was more pronounced on aged muscles and did not fully recover at 6 weeks post sciatic nerve crush. Of note, at day 6 after crush, when no change in muscle volume was yet detected by MRI, a significant increase of T_2_ could be seen in the calf muscle, consistent with increased extracellular space due to type IIb fiber atrophy as revealed by histology (Giorgetti et al., 2019).

For consistency, the approach was applied to another demyelination model, in which lysolecithin was injected upon the sciatic nerve of rats. The same spatial pattern of MTR reduction observed for the nerve crush was reproduced, with the most pronounced MTR changes occurring at the bifurcation of the sciatic nerve. The reduced MTR was accompanied by reductions in luxol fast blue staining detected histologically (Giorgetti et al., 2019).

Although so far only used to phenotype the models, it remains to be demonstrated that MRI will be useful when assessing therapies. The fact that MTR was sensitive to detect effects of compounds aiming to improve myelination in the brain (Beckmann et al., 2018; Dietrich et al., 2022; see below) indicates that this might also be possible in the peripheral nervous system. In comparison to electrophysiological assessments, MRI has the advantage of providing spatial information. Moreover, the temporal evolution of nerve conduction and MTR assessments are not necessarily the same, as mentioned for old mice in the nerve crush experiment. In addition to peripheral nerves, MRI can also analyze the central nervous system of the animals. Besides MTR, diffusion tensor imaging (DTI) providing metrics such as fractional anisotropy, axial diffusivity, radial diffusivity, and mean diffusivity, is also an alternative to analyze peripheral nerve dysfunction and repair (Kim et al., 2019; Cheah et al., 2021). However, the demanding technical nature of DTI requiring specialized expertise and long measurement times (12 h were reported for excised rat nerves; Boyer et al., 2015) might be challenges for its routine application to preclinical pharmacological examinations in small rodents. Finally, MTR has been established in the clinic as reliable and reproducible (Preisner et al., 2021; Chen et al., 2023) and provides a means to examine peripheral nerve injury and neuropathies (Dortch et al., 2014; Kollmer et al., 2020; Roth et al., 2022), thereby enhancing the translational potential of the activities described here for small rodents.

### Brain

#### Neurodegeneration in multiple sclerosis

**FIGURE 3:**
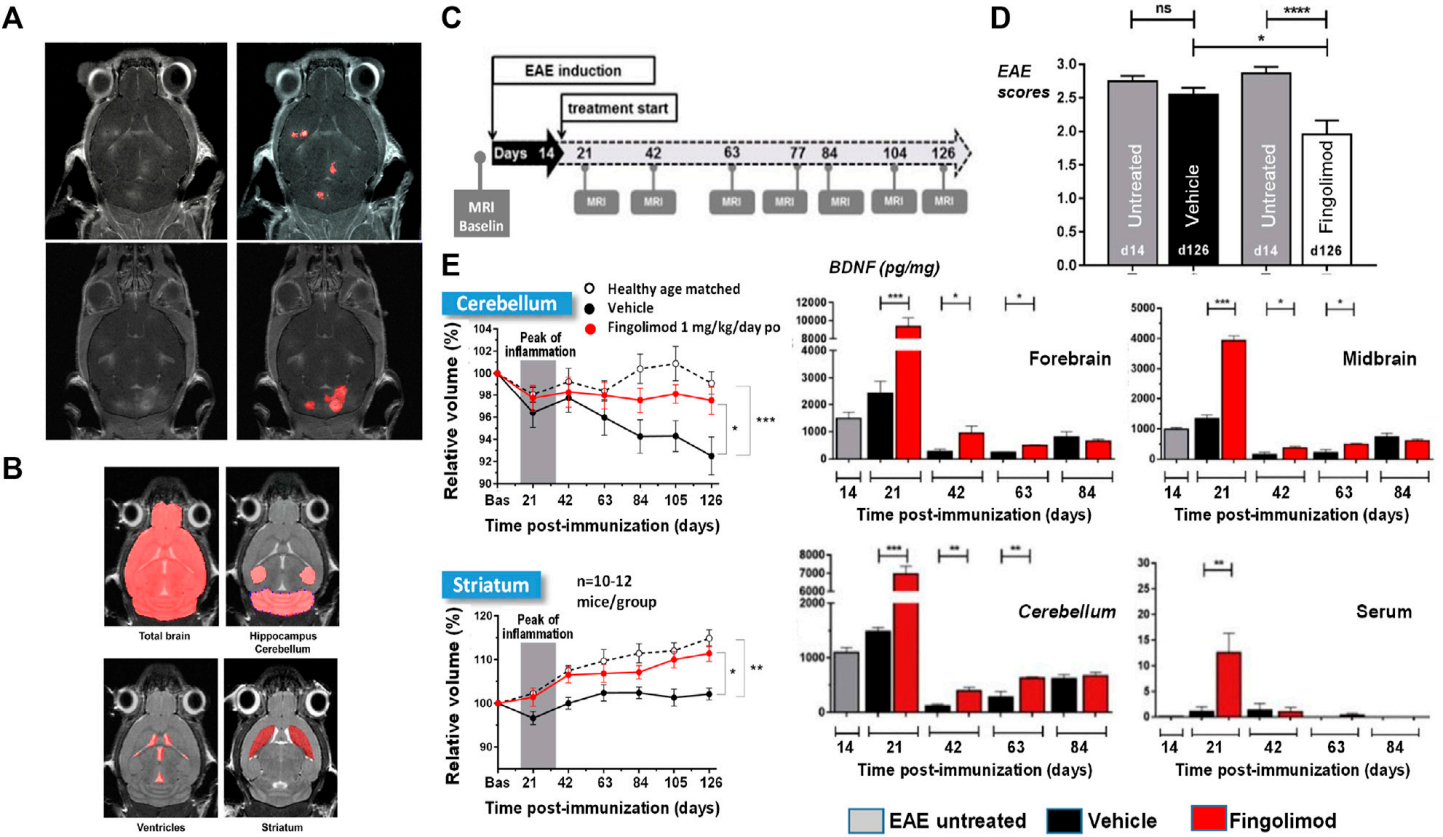
MRI for the analysis of EAE mice modeling MS: Lesion detection and neurodegeneration. **(A)** T_1_-weighted images acquired at the peak of disease from two EAE animals following intravenous injection of the clinically approved contrast agent Dotarem (Gd-DOTA) as a bolus. Lesions corresponding to leakage of the contrast agent in areas of impaired blood-brain barrier are clearly visible. **(B)** For volumetric analyses of the whole brain and subareas thereof T_2_-weighted images acquired in 12.5 min without administration of contrast material provided sufficient contrast for segmentation. **(C)** Scheme of study protocol for assessing neurodegeneration in the model. Treatment started at the peak of disease on day 14 after EAE induction. **(D)** EAE mice treated with fingolimod had improved clinical scores compared to animals receiving vehicle. **(E)** Neurodegeneration was consistently quantified by MRI in the striatum and cerebellum of EAE mice receiving vehicle but not in those treated with fingolimod. Increased brain derived neurotrophic factor (BDNF) levels were detected in several brain areas and in the serum of fingolimod-treated EAE mice. More details can be found in Smith et al. (2018). ^©^ 2018 Elsevier B.V.

Multiple sclerosis (MS) is the most common non-traumatic disabling disease affecting young adults (Browne et al., 2014). Although historically considered as an organ-specific T-cell mediated autoimmune disease, recently the involvement of also B-cells in MS became clearly evident (Greenfield and Hauser, 2018). The disease is viewed as having two stages, comprising early inflammation responsible for a relapsing–remitting pattern and delayed neurodegeneration causing non-relapsing progression in secondary and primary progressive MS (Leray et al., 2010; Thompson et al., 2018). Therapies for MS are required to reduce the number of relapses and to lead to less disability as well as brain lesions detected by gadolinium-MRI (Figure 3A). Moreover, slowing brain atrophy has become a key clinical efficacy readout (Guevara et al., 2019).

Described for several species, experimental autoimmune encephalomyelitis (EAE) is a common animal model in preclinical MS research (Baker and Amor, 2014). In mice, it involves the subcutaneous administration of myelin oligodendrocyte glycoprotein in complete Freund adjuvant, boosted the intraperitoneal injection of pertussis toxin. Brain inflammation, demyelination and neurodegeneration are observed.

Although important for molecular characterization and target validation studies in this model, histology has its limitations when it comes to pharmacological studies. Besides necessitating large number of animals and not being applicable in the clinic, it is very time consuming especially for deriving volumetric information, and it is prone to sampling errors, as brain volume changes (shrinkage) may occur at autopsy. The feasibility of detecting brain volumetric changes of less than 10% in EAE mice by MRI, using a field strength of 7 T and a conventional radiofrequency coil, has been demonstrated (MacKenzie-Graham et al., 2012). However, acquisition times of the order of 1 h or longer were necessary.

Knowing that EAE mice are very susceptible to their environment, we aimed at having a short measurement time. Following optimization the whole brain was imaged with sufficient contrast for quantifying subareas in a reasonable measurement time of 12.5 min without administration of contrast material (Smith et al., 2018) (Figure 3B). In two separate preparatory studies it was verified whether repeated use of the 12.5 min protocol impacted the disease development, assessed through scores of limb paralysis. Only when we showed that no impact occurred, did pharmacological testing start.

A compound tested in the model was the sphingosine 1-phosphate (S1P) receptor agonist, fingolimod (Smith et al., 2018). Treatment started at the peak of the disease, at day 14 after EAE induction, and went until the end of the study, on day 126 (Figure 3C). MRI was performed at baseline and at different time points after EAE induction. Fingolimod improved the clinical scores of the EAE animals (Figure 3D). MRI revealed that the cerebellum volume decreased with time in EAE mice receiving vehicle. Moreover, both the cerebellum and the striatum volumes in vehicle-treated EAE mice were significantly lower compared to those in normal mice which served as controls. These observations were consistent with neurodegeneration occurring in the model. Moreover, the longitudinal development of the cerebellum and striatum volumes of fingolimod-treated EAE mice followed the same pattern as that observed for normal mice, showing that the compound protected against neurodegeneration in EAE mice (Figure 3E). Studies performed on other cohorts showed that brain derived neurotrophic factor (BDNF) was increased in several brain areas at least until day 63, as well as in plasma in the initial phase (Figure 3E), providing a possible mechanism for the neuroprotective effects of fingolimod.

In the clinic, several studies showed that fingolimod slowed down neurodegeneration in MS patients as assessed by MRI (Kappos et al., 2015; Zivadinov et al., 2018). Moreover, increased BDNF secretion from circulating T-cells was detected in MS patients receiving the compound (Golan et al., 2019).

MRI opens the avenue for performing neurodegeneration studies in the EAE model. From the 3Rs perspective, besides an impressing reduction of animal numbers (estimated reduction 88%), using a clinically relevant imaging approach improves the translational validity of the MS animal model for compound testing.

#### Demyelination in the central nervous system: Cuprizone model

Various disorders of the central nervous system, including leukodystrophies and genetic disorders as hematoidosis, Niemann-Pick’s disease and aminoacidopathies, are characterized by either demyelination or the destruction of a previously intact myelin sheath. However, MS is the most frequent neurological disease involving myelin pathology. Therapies targeting remyelination have a great potential to delay, prevent or even reverse disability in MS patients.

**FIGURE 4:**
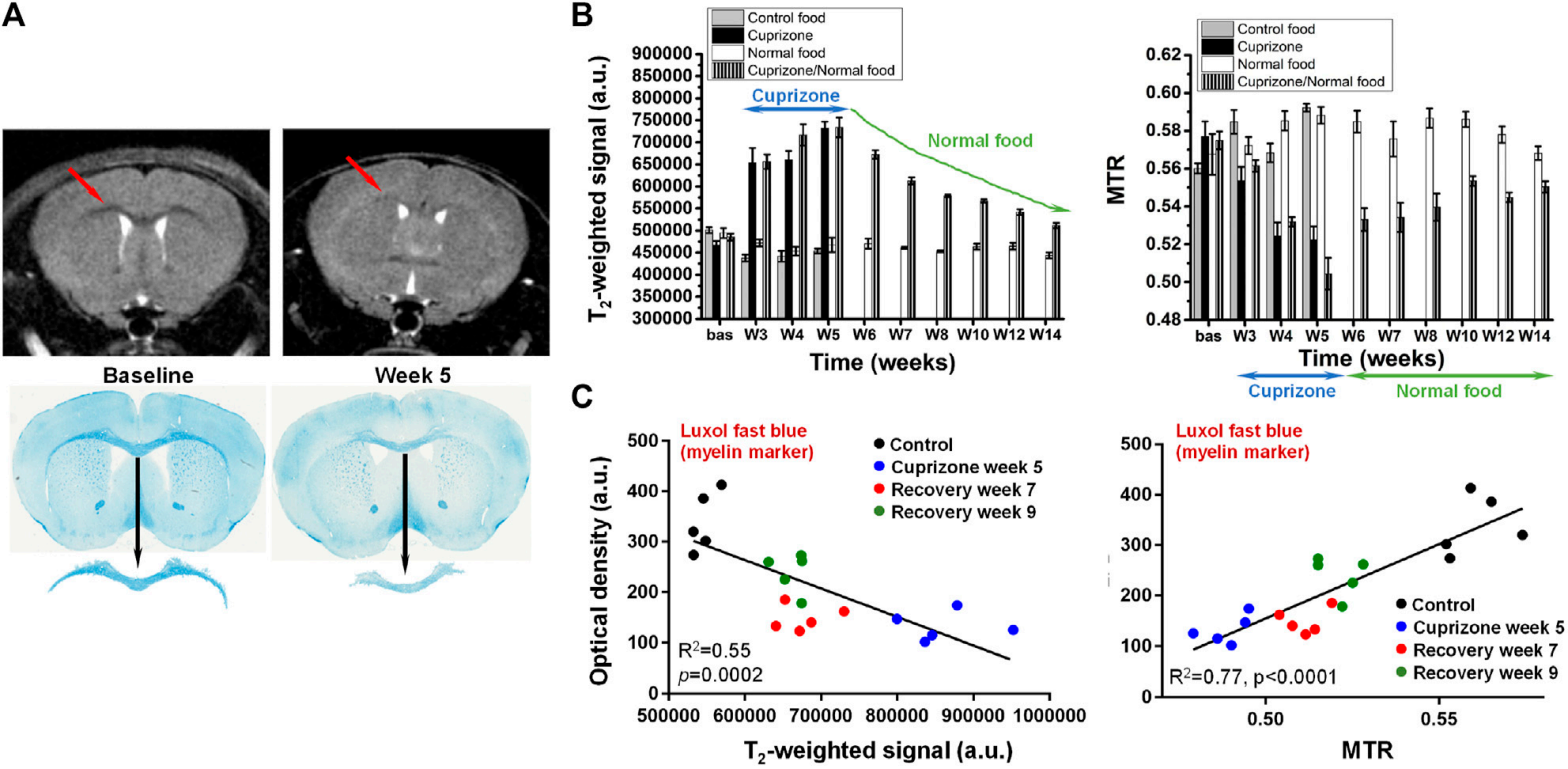
Cuprizone-induced demyelination in the brain of mice. **(A)** Representative T_2_-weighted images from the same mouse acquired before (baseline) and after 5 weeks of cuprizone intoxication. A clear contrast change occurred at the level of the corpus callosum (arrows). Luxol fast blue histology revealed demyelination in the same brain area. **(B)** Cuprizone induced significant signal increase in T_2_-weighted images respectively decrease of magnetization transfer ratio (MTR) in the corpus callosum. **(C)** Comparison between the MRI signal/MTR parameters and the quantitative histology analysis of luxol fast blue. More details can be found in Beckmann et al. (2018). ^©^ The Authors. 2018 Open Access.

The cuprizone model, involving toxin-induced demyelination followed by endogenous remyelination after cessation of the intoxication, is largely used to test the efficacy of novel compounds *in vivo* (Torkildsen et al., 2008). It has also contributed substantially to the understanding of important aspects of the MS disease. MRI is an ideal tool to follow longitudinally and non-invasively the pathology in this mechanistic model. Contrast changes in T_2_-weighted images became clearly apparent in the corpus callosum of mice receiving the copper chelator in food pellets for 5 weeks, which were then reverted after the interruption of cuprizone administration (Figure 4A). Histology of the myelin marker, luxol fast blue, clearly demonstrated less myelin in the corpus callosum at week 5. A summary of the T_2_-weighted signal in this brain area is provided in Figure 4B. For the same animals, a reduction of MTR occurred during the cuprizone intoxication phase. Upon interruption of cuprizone, MTR slowly increased towards baseline values. Signal intensity correlated negatively, while MTR correlated positively with histology, either with luxol fast blue, a myelin marker, or with myelin oligodendrocyte glycoprotein (Figure 4C).

In pharmacological studies involving the colony-stimulating factor-1 inhibitor, BLZ945, administered preventively, before and during the cuprizone intoxication, both T_2_-weighted signal and MTR suggested that the compound had some protective effects again demyelination in the corpus callosum, but less in the external capsule (Beckmann et al., 2018). This was confirmed by luxol fast blue histological analysis. Mice receiving BLZ945 and cuprizone treatment for 5 weeks displayed in the corpus callosum a substantial amount of remaining myelin, and a reduction of Iba1-positive microglia. When BLZ945 was administered therapeutically, namely, starting at week 5 of cuprizone intoxication, T_2_-weighted signal in the cortex and striatum was normalized, suggesting increased remyelination in these brain areas. However, no effect of the compound was detected in the corpus callosum. Histology confirmed the *in vivo* MRI observations (Beckmann et al., 2018).

In secondary progressive MS patients, siponimod, a selective S1P receptor 1 and 5 modulator, significantly reduced disability progression, cognitive decline, and total brain volume loss compared to placebo treatment (Kappos et al., 2018; Regner-Nelke et al., 2022). Some of these protective effects might be modulated by the induction of remyelination. Evaluations of MTR and T_2_-weighted signals revealed indeed increased remyelination in the cuprizone model for mice treated with siponimod (Dietrich et al., 2022).

Microglia, osteoclasts, dendritic cells and macrophages express the cell-surface immunoreceptor TREM2 (triggering receptor expressed on myeloid cells 2) (Jay et al., 2017). Myelin/neuronal loss and neuroinflammation in neurodegenerative diseases like Alzheimer`s disease and frontotemporal dementia have been associated with heterozygous loss-of-function TREM2 mutations, most notably those enhancing cell-surface shedding (Jay et al., 2017; Yeh et al., 2017). The role of soluble and cleavage-reduced TREM2 on myelination processes in the brain has been investigated in TREM2 cleavage-reduced, TREM2 soluble-only, TREM2 knock-out and wildtype mice analyzing MRI readouts within the cuprizone model (Beckmann et al., 2023). Upon cuprizone challenge sustained microglia activation led to increased remyelination, whereas microglia with only soluble TREM2 had reduced phagocytic activity despite displaying an efficient lysosomal function, resulting in a dysfunctional phenotype comprising impaired myelin debris removal capacity, lack of remyelination and axonal pathology.

Although most cuprizone studies define the corpus callosum as main region of interest for evaluations, demyelination also occurs in other white and gray matter areas (Goldberg et al., 2015). MRI provides the opportunity to analyze simultaneously several brain areas, which is certainly an advantage in pharmacological studies using the model. Since some variability can be expected in response to cuprizone, for therapeutic treatment starting after some weeks of intoxication, randomization of animals into different groups based on MRI just before initiation of compound dosing becomes of paramount importance. Acquisition of T_2_-weighted and MTR images for every animal enabled to also derive information on myelin debris (Beckmann et al., 2018; 2023), an important aspect when testing compounds as the presence of debris may impair remyelination (Lubetzki et al., 2020). Moreover, there is translational potential of the MRI readouts. Hyperintense lesions on T_2_-weighted images of MS patients are considered as a sign of demyelination in the central nervous system (Thompson et al., 2018) and MTR has been demonstrated to be sensitive to cortical demyelination in MS patients (Chen et al., 2013).

#### Brain function

The power of functional MRI (fMRI) to study brain disease and pharmacology has been reviewed extensively elsewhere (Bifone and Gozzi, 2012; Jenkins, 2012; Jonckers et al., 2015; Carmichael et al., 2018). Functional experiments can be classified into: 1) Task-based fMRI employing sensory or cognitive stimuli to induce responses in brain regions or circuits; 2) resting-state fMRI (rs-fMRI) used to investigate functional connectivities in the absence of any stimulus; 3) pharmacological MRI (phMRI) dealing with fMRI signals after the administration of pharmacological agents, with the aim to localize the target area in the brain containing the appropriate receptors for the neuromodulatory agents. Upon neural activation, changes in local cerebral blood flow and volume as well as in the cerebral metabolic rate of oxygen lead to a locally increased ratio of oxygenated over deoxygenated hemoglobin. These mechanisms provide the basis for fMRI, which relies primarily on the acquisition of images that are sensitive to the blood level dependent (BOLD) contrast based on the differential magnetic properties of oxygenated (diamagnetic) and deoxygenated (paramagnetic) hemoglobin or to perfusion as assessed using, e.g., arterial spin labeling techniques. Increased ratios of oxygenated over deoxygenated hemoglobin with neural activation results in contrast changes, for instance in a local signal enhancement in T_2_^∗^-weighted images. Despite providing only an indirect measure of neuronal activity, fMRI is a powerful tool to examine brain function, as attested by the large number of clinical trials using fMRI as an outcome measure (Sadraee et al., 2021).

The translational character of fMRI between rodents and humans has been carefully addressed by several groups. For instance, robust responses upon ketamine dosing were detected in the cingulate, frontal cortex, and hippocampus (Bifone and Gozzi, 2012) and acute ketamine challenge increased the resting state prefrontal-hippocampal connectivity in both humans and rats (Grimm et al., 2015), a phenotype that is often disrupted in pathological conditions related to psychiatric disorders and their onset. A good agreement between phMRI signatures in rodents and humans was also shown for acute remifentanil administration, with activation present in the striatum, thalamus, hippocampus, and cingulate cortex (Leppä et al., 2006; Liu et al., 2007). Amphetamine as well induced correlated responses in the reward circuitry in both species (Völlm et al., 2004; Schwarz et al., 2007). The default-mode network, initially observed in humans (Raichle et al., 2001) and nonhuman primates (Vincent et al., 2007), has likewise been measured using rs-fMRI techniques in the rat and mouse brain (Lu H. et al., 2012b; Stafford et al., 2014; Gozzi and Schwarz, 2016). Other networks, such as the striatal system, were detected in the rodent brain as well (Becerra et al., 2011; Jonckers et al., 2011; Bajic et al., 2017; Grandjean et al., 2017).

As mentioned previously, fMRI covers multiple paradigms, each of which may differ in implementation details and performance characteristics. Recommendations and good practices for fMRI studies were summarized by different groups (Schwarz et al., 2011a; Schwarz et al., 2011b; Mandeville et al., 2014; Khalili-Mahani et al., 2017). Performing brain fMRI studies in small rodents poses additional challenges, related not only to data acquisition and analysis but also to anesthesia (Pan et al., 2015; Chuang and Nasrallah, 2017; Sumiyoshi et al., 2019; Huang et al., 2022). Although patterns of resting-state functional connectivity have been shown to be present in humans under anesthesia (Greicius et al., 2008) or during the early stages of sleep (Larson-Prior et al., 2009), great care needs to be taken when performing brain functional in anesthetized small rodents. Multiple anesthesia regimens were tested (see, e.g., Bukhari et al., 2017; Wu et al., 2017). The use of low doses of isoflurane or medetomidine has been reported in small rodent fMRI studies. By combining both agents vasodilatory effects of isoflurane, resulting in a dose-dependent increase of cerebral blood flow that influences neurovascular interactions detected by fMRI, may thus be counteracted by medetomidine, which is known to dose-dependently cause vasoconstriction (Nakai et al., 1986). For fMRI studies in mice, intubation, artificial ventilation and even paralysis with pancuronium bromide has been sometimes adopted (Bukhari et al., 2017; Wu et al., 2017; Pagani et al., 2021). Of note, mechanical ventilation may sometimes inadvertently cause lung injury (Walder et al., 2005; Nickles et al., 2014), especially if applied repeatedly. Awake animal imaging is also an alternative, as demonstrated by the robust and reproducible detection of brain networks in conscious rats and mice (Becerra et al., 2011; Liang et al., 2011; Liu et al., 2020; Fadel et al., 2022; Gutierrez-Barragan et al., 2022), but has its own constraints related to motion, stress and habituation.

Given the complexity of brain fMRI studies, collaborative work of various groups aiming at standardizing acquisition/analysis protocols is promoting the dissemination and reuse of clinical data (Alfaro-Almagro et al., 2018; Esteban et al., 2019; Notter et al., 2022). In the preclinical area, acquisitions in animals have been reported using a multitude of protocols comprising differences in strains, anesthesia conditions, coil designs, magnetic fields and data analysis pipelines, to name a few distinctive features. In analogy to the clinics, collaboration between several labs around the globe were also reported recently for fMRI acquisitions in animals (Mandino et al., 2020; Grandjean et al., 2020; Grandjean et al., 2023). Dissemination and comparison of learnings/experience through such consortia might lead to optimized consensus protocols that could substantially facilitate future experimental work in this area.

### Lung

A short overview of imaging techniques of interest for pharmacological research in pulmonary diseases has been provided elsewhere (van Echteld and Beckmann, 2011; Beckmann and Crémillieux, 2016). Here, we illustrate how MRI can be used to refine models of pulmonary fibrosis and cancer in small rodents.

#### Bleomycin-induced lung injury

Pulmonary fibrosis is characterized by the accumulation of inflammatory cells, excessive fibroblast proliferation, increase in collagen content, and deposition of extracellular matrix in the lungs (Strieter and Mehrad, 2009; King Jr et al., 2011). The local administration of bleomycin into the lungs is commonly used to model pulmonary fibrosis in small rodents, resulting in a phenotype that mimics in many respects the human disease (Jenkins et al., 2017). MRI provided the opportunity to non-invasively follow the course of bleomycin-induced lung injury in mice (Babin et al., 2012; Egger et al., 2013) and rats (Karmouty-Quintana et al., 2007; Jacob et al., 2010; Babin et al., 2011; Egger et al., 2013), with acquisitions performed in spontaneously breathing animals without respiratory gating. Micro-CT has also been adopted to detect fibrosis-related lesions in bleomycin models (Ask et al., 2008; De Langhe, et al., 2012). Considering that the lung tissue is particularly sensitive to cumulative doses of ionizing radiation (Plathow et al., 2004; Graves et al., 2010; Tang et al., 2020), it needs to be kept in the mind that radiotoxicity may play a role for repeated micro-CT scanning. The excellent agreement found between *in vivo* MRI, *in vivo* micro-CT and standard histological measures of lung fibrosis in mice (Velde et al., 2014) provides clear evidence in favor of MRI as imaging readout in the bleomycin model.

**FIGURE 5:**
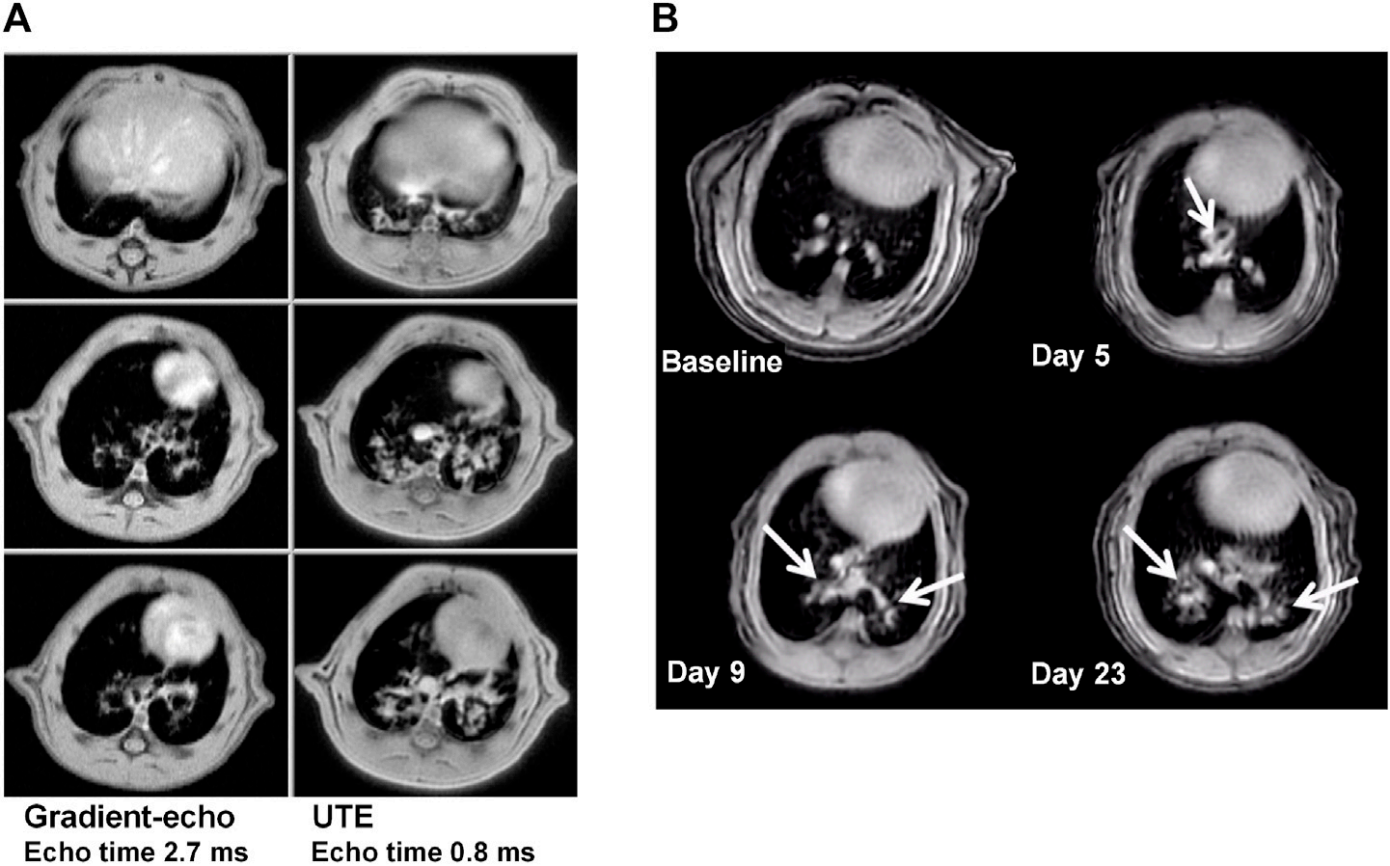
MRI at 4.7 T in the bleomycin model, for animals under spontaneous respiration. **(A)** Comparison between two-dimensional gradient-echo and UTE images acquired in 22 and 7.4 min, respectively, from one Sprague Dawley rat in the same imaging session at day 15 after bleomycin challenge (4 mg/kg intra-tracheal). The three slices for each acquisition method correspond to the same anatomical location. Note the increase in sensitivity for lesion detection by using ultrashort echo time technique. **(B)** Detection of bleomycin-induced lung injury by UTE-MRI in a BALB/c mouse. Comparable slices from two-dimensional UTE images (4 min acquisition time, echo time 0.5 ms) before and at different timepoints after oropharyngeal bleomycin administration (1.0 mg/kg/day on 6 consecutive days). Bleomycin-elicited lesions are indicated by the arrows. See Egger et al. (2013), Egger et al. (2014) for more details. ^©^ 2013 Egger et al. and ^©^ 2014. The American Physiological Society.

While gradient-echo MRI was initially employed for the quantification of bleomycin-induced injury (Karmouty-Quintana et al., 2007; Babin et al., 2011; Babin et al., 2012), introduction of ultrashort echo time (UTE) acquisitions improved the sensitivity for detecting lesions (Figure 5A). This increased sensitivity had the beneficial consequence of a reduction in measurement times by factors of 3–5, with two-dimensional UTE acquisitions declining respectively to 7.3 min and 4 min for rats and mice (Egger et al., 2013) (Figure 5B). Three-dimensional UTE with an echo time of 20 µs enabled images of the lungs to be acquired at higher spatial resolution in 11.6 min and 6.9 min for rats and mice, respectively (Egger et al., 2014). Also, the bleomycin dose could be reduced, providing another experimental refinement.

Fibrotic process can lead to an impairment of lung function reflected in changes in tidal volume and breathing cycle times as shown for mice challenged with bleomycin (Milton et al., 2012). Also, increased lung elastance and reduced compliance occur in pulmonary fibrosis models (Ask et al., 2008; Manali et al., 2011). An increase of total lung volume, consistent with increased post-mortem dry and wet lung weights, hydroxyproline content as well as collagen level, was determined *in vivo* by MRI in bleomycin animals (Egger et al., 2014). Respiration-gated MRI demonstrated an increased lung volume at both inspiration and expiration, as well as a transient decrease of the tidal volume for bleomycin-treated rats. Terminal lung function analyses performed in tracheotomized and mechanically ventilated bleomycin rats using a flexyVent^®^ system revealed decreased dynamic lung compliance (Egger et al., 2014). In summary, the increase of lung volume quantified by MRI after bleomycin administration was in agreement with tissue remodeling accounting for a reduced lung elasticity. Therapeutic treatment of bleomycin rats with the somatostatin analogue, SOM230, resulted in a decrease of lesion and total lung volume, the latter observation suggesting an improvement of lung function in the diseased animals (Egger et al., 2014).

From the standpoint of animal welfare, many factors contribute to a refinement of the fibrosis experiments introduced by MRI: measurements performed in spontaneously respiring animals; high sensitivity to detect lesions using UTE enable fast acquisitions and/or a reduction of bleomycin dose; quantification of changes in total lung volume allow functional information reflecting reduced lung elasticity due to fibrosis development to be derived; MRI constitutes an imaging alternative free from ionizing radiation for assessing fibrosis in small rodent models. These features are of relevance in pharmacological studies, particularly when considering therapeutic effects of compounds administered when fibrosis is already established in the lungs. MRI has indeed been incorporated into several preclinical drug investigations and in the *in vivo* validation of pharmacological targets involving the bleomycin model (Babin et al., 2011; Babin et al., 2012; Egger et al., 2014; Egger et al., 2015a; Egger et al., 2017).

#### Lung tumor resistance

In recent years, the generation of animal models has been facilitated by CRISPR as well as developments around *in vivo* gene delivery technologies, including viral vectors, electroporation, and lipid nanoparticles (Ciampricotti et al., 2021; Lima and Maddalo, 2021). The combination of specific delivery methods optimized to the organ of interest with the CRISPR/Cas9 system has enabled consistent genome editing of somatic cells at the target organ (Maddalo et al., 2014). Complex genetically engineered mouse models could thus be generated relying on the versatility of CRISPR/Cas9 (Maddalo et al., 2014). Moreover, a substantial reduction in animal numbers were achieved because in the generation of these so-called somatically engineered mouse models, germline engineering and animal breeding steps are skipped.

**FIGURE 6:**
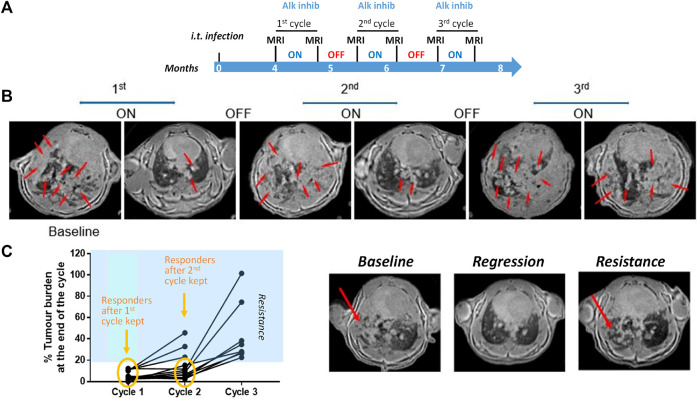
Imaging in a mouse lung tumor model. **(A)** Study protocol scheme. **(B)** MRI images from one representative mouse at different time points of the treatment phases. Tumor lesions are indicated by the red arrows. **(C)** Summary of tumor burden based on the quantification of lung lesions in the images. Images from one mouse acquired at baseline (left), at the end of the first (middle) and third cycle of treatment (right). See Egger et al. (2013) for details on image acquisition and lesion quantification. ^©^ 2013 Egger et al.

This section exemplifies studies around tumor resistance in a CRISPR-induced mouse model of anaplastic lymphoma kinase (Alk) positive non-small cell lung cancer (NSCLC), for which adenoviral particles expressing the CRE recombinase and the CRISPR/Cas9 system were administered intra-tracheally. The study protocol is summarized in Figure 6A: 4 months after the infection, MRI was performed to quantify the tumors in the lungs. A first 3-week-cycle of treatment with an Alk inhibitor followed. MRI was performed again to verify the effect of the first cycle of treatment. The next 3 weeks were of treatment free, and at the end of this period MRI was again performed. This was repeated twice. MRI images for one representative mouse are shown in Figure 6B. The MRI baseline at 4 months after infection showed that the lung was full of lesions. Following treatment with the Alk inhibitor, these lesions were wiped off. However, the cancer lesions reappeared after the treatment was interrupted. A second cycle of treatment reduced the lesions, but this time, some of them persisted. Following treatment interruption, the lung was again full of lesions. A third round of Alk inhibitor treatment was definitely much less efficient compared to the previous ones, suggesting resistance of the lung cancer to treatment. This is summarized for a number of mice in Figure 6C.

In the present example, an estimated reduction of animal usage by 90% was achieved by using CRISPR, and this number increased to 98% by the incorporation of MRI in the experiment. Although micro-CT could also have been used as imaging modality, the effects of repeated exposure to ionising radiation in particular in tumor models needs to be taken into account. Recently, MRI and CT were shown to be equivalent to monitor lung cancer-bearing mice, but non-ionising MRI was considered particularly well suited for longitudinal studies (Spiro et al., 2020; Baier et al., 2020; Baier et al., 2023). Finally, chromosomal rearrangements of Alk are detected in 3%–7% of NSCLCs (Soda et al., 2007) and lung cancers displaying Alk rearrangements are highly sensitive to Alk tyrosine kinase inhibition. However, despite a high response rate of 60% in Alk-rearranged NSCLC, resistance to therapy based on Alk inhibition was shown to develop typically within one to 2 years (Friboulet et al., 2014; Mizuta et al., 2021). The model discussed here may be helpful when testing new therapies to overcome resistance.

### Liver

#### Liver regeneration

The ability of the liver to regenerate itself upon loss of hepatic tissue has been known for a long time. However, reduced regenerative capacity exists under various circumstances, for instance in chronic liver disease, acute liver failure or liver resection encompassing tumor surgery. Methods that enhance the intrinsic regeneration potential of the liver are therefore required. Hepatic cell line-based *in vitro* systems Molecular mechanisms of liver cell growth can be studied in hepatic cell line-based *in vitro* systems. On the other hand, complex processes such as liver development or regeneration need to be studied *in vivo*. For this purpose, partial hepatectomy performed in small rodents, consisting of the surgical removal of three of the five liver lobes, is often adopted. Cells of the remaining two lobes proliferate and a complete recovery with the liver regaining its original size is achieved within approximately 8 days after surgery (Mitchell and Willenbring, 2008).

Taking animal welfare into account, noninvasive imaging has a role to play when it comes to assess liver regeneration longitudinally. Two important parameters can be quantified by imaging, namely, the pre-surgery liver volume and the amount of actually excised tissue. For instance, Miyazaki et al. (2011) employed x-ray micro-CT to assess the regenerating direction and the shape of the regenerated remnant liver in hepatectomized rats (Miyazaki et al., 2011). Radiation dose may be a limiting factor in view of repeated scans within a relatively short time interval when CT-based liver volumetry is applied to small rodents. Such concerns obviously do not exist for MRI, which has also been demonstrated to allow precise liver volume determination before and following partial hepatectomy in mice (Garbow et al., 2004; Inderbitzin et al., 2004) or rats (Hockings et al., 2002) without or with application of contrast material. To reduce movement artifacts, respiration gating was used, and acquisition times ranged from 7 to 42 min.

MRI volumetry has also been established as a primary end point of liver regeneration in a murine model of partial hepatectomy with the scope of performing pharmacologic experiments (Orsini et al., 2016). Acquisitions of 14.5 min duration performed on anesthetized, spontaneously respiring animals, without gating and administration of contrast agent resulted in a highly significant correlation (R = 0.98, *p* = 1.5 × 10^−14^) between the MRI-derived liver volumes and the post-mortem liver weights in hepatectomized, untreated mice. 1,4-bis [2-(3, 5-dichloropyridyloxy)] benzene (TCPOBOP), a synthetic agonist of the mouse constitutive androstane receptor and a potent activator of cytochrome P450 monooxygenase activity (Halwachs et al., 2007) shown earlier to induce hepatocyte proliferation and hepatomegaly (Ledda-Columbano et al., 2000), was then evaluated as test compound in the model. An enhanced liver regrowth capacity upon TCPOBOP treatment was revealed *in vivo* by MRI and confirmed by *post mortem* comparative hepatocyte proliferation assays (Ki67 expression) and liver weight analysis (Orsini et al., 2016). The feasibility of using imaging in pharmacologic studies in the context of liver regeneration has thus been demonstrated. In comparison to terminal procedures, the number of hepatectomized mice needed to derive a liver (re)growth curve was reduced by a factor of 6. Following this validation step, imaging was also included in studies demonstrating that the RSPO-LGR4/5-ZNRF3/RNF43 module controls metabolic liver zonation and constitutes a hepatic growth/size rheostat during development, homeostasis and regeneration (Planas-Paz et al., 2016).

In the clinic, liver volumetry is important in the context of liver resection and transplant surgery. Partial hepatectomy has become an important approach to address many primary and secondary hepatic tumors (Orcutt and Anaya, 2018; Riddiough et al., 2021). However, the percentage of functional liver parenchyma remaining after major hepatic resection is crucial to predict surgical success (Kishi et al., 2009). Imaging-based liver volumetry demonstrated that measurable changes in remnant liver volume begin approximately 5 days following surgery (Simpson et al., 2014). Also, imaging is important in the domain of living donor liver transplantation, both for the preoperative evaluation of the donor liver (Kim et al., 2018) and for the assessment of remnant liver regeneration in the follow-up of donors (Klink et al., 2014). Deep learning applied to MRI data is gaining attention due to the precision achieved in segmental volume assessments (Mojtahed et al., 2022).

#### Nonalcoholic steatohepatitis (NASH)

Non-alcoholic fatty liver disease (NAFLD) is one of the most common liver disorders, in which hepatic steatosis occurs in the absence of secondary causes like medications, excessive alcohol consumption, or heritable conditions (Byrne and Targher, 2015; Friedman et al., 2018). Around 25% of the world population is estimated to have NAFLD, and 25% of NAFLD patients are thought to have nonalcoholic steatohepatitis (NASH) (Younossi et al., 2018), characterized by excessive liver fat accumulation, hepatic inflammation and fibrosis (Diehl and Day, 2017; Sheka et al., 2020). Often clinically silent, with time NASH can progress to cirrhosis, end-stage liver disease, and ultimately the need for an organ transplant.

Changes of dietary habits and exercise aiming to reduce weight constitute the basis of NASH treatment, and no specific therapies do exist. Early detection is a prerequisite to control the impact of the condition, the challenge being the frequently asymptomatic nature of NASH, as mentioned before. Patients presenting high body mass index (>25 kg/m^2^) and type 2 diabetes mellitus features comprising hyperglycemia and insulin resistance are recommended to test for fatty liver disease (Friedman et al., 2018). Although elevation of the liver enzymes alanine transaminase (ALT) and aspartate aminotransferase (AST) in the blood plasma generally provides a first line of evidence (Wong et al., 2018), analysis of a liver biopsy is the gold standard for assessing the presence and severity of NASH.

Since biopsies entail a small risk of complications such as bleeding and represent only a small fraction of the liver volume, often resulting in an underestimation of disease severity, imaging alternatives are of need. Indeed, MRI and ultrasound can be applied for the noninvasive assessment of fat and fibrosis in the liver (Ajmera and Loomba, 2021). For instance, MRI-derived proton density fat fraction (MRI-PDFF) provides an accurate measure of liver fat content and has been adopted in early-phase NASH trials (Caussy et al., 2018). Ultrasound relying on the assessment of parameters such as the attenuation, backscatter coefficient, and speed/wavelength of the ultrasonic wave can be used as well for the quantification of hepatic steatosis (Han et al., 2020). Elastography based on MRI or shear wave ultrasound provides liver stiffness measurements as a surrogate quantitative biomarker for fibrosis (Xiao et al., 2017; Ozturk et al., 2022).

Currently established animal models of NASH are broadly divided into three main categories: dietary-induced, diet-toxin-induced, and diet-genetically mutated models (Farrell et al., 2019; Peng et al., 2020). Dietary regimens comprise high fat diet, methionine deficient diet, choline deficient diet, methionine choline deficient diet, amylin NASH diet, or high fat diet containing cholesterol supplemented by high fructose and sucrose. Toxins such as streptozotocin, diethylnitrosamine or carbon tetrachloride (CCl_4_) can be added to the diet to increase the severity of liver injury. Genetic models include leptin-receptor-deficient mice, apolipoprotein E knock out mice, 148 isoleucine to methionine protein variant (I148 M) of patatin-like phospholipase domain-containing protein 3 (PNPLA3) knock-in mice, or mice overexpressing urokinase plasminogen activator introduced into hepatocytes via adeno-associated virus.

**FIGURE 7:**
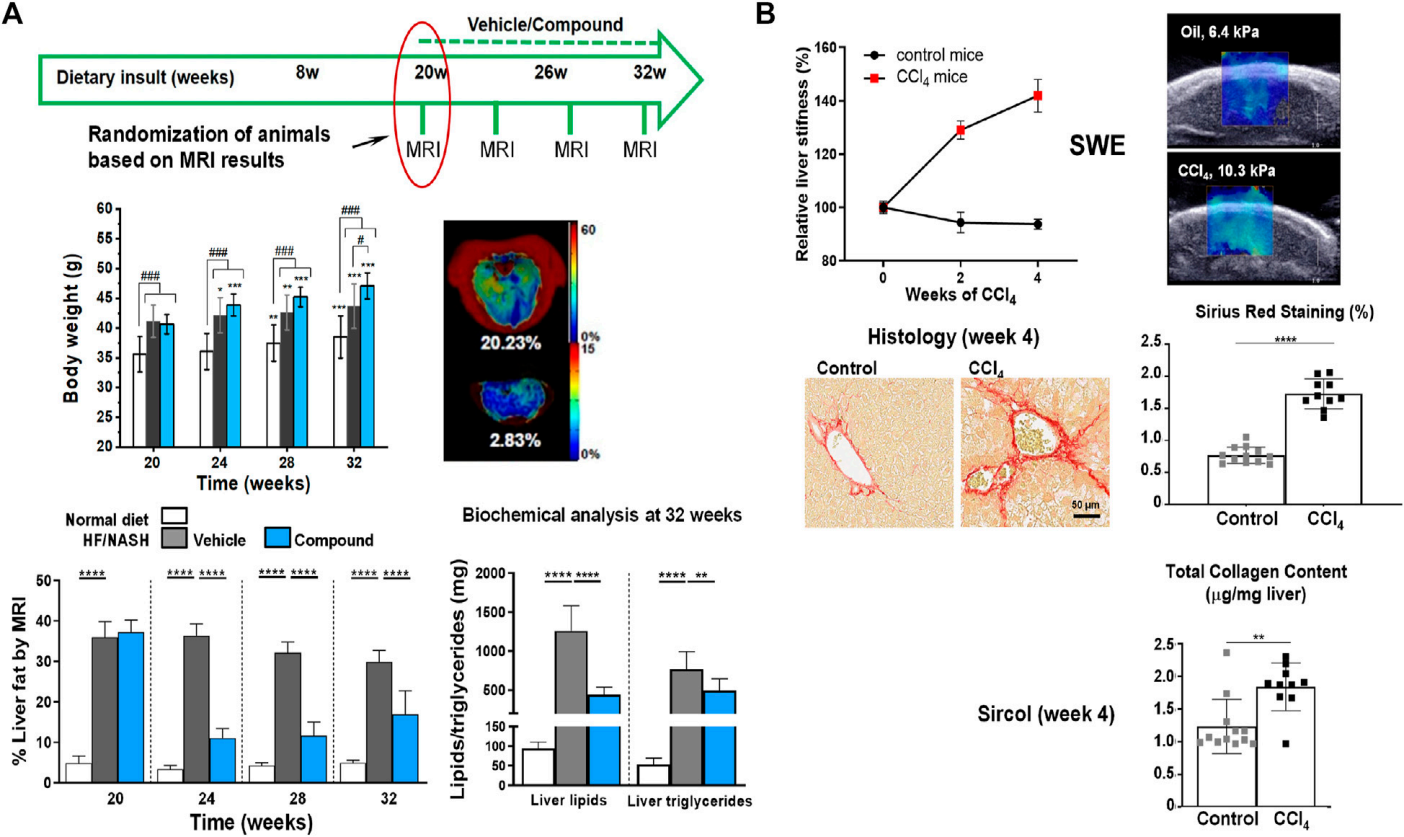
Imaging analysis of liver injury in mouse models. **(A)** Longitudinal non-invasive liver fat quantification by MRI. Livers of mice receiving a NASH diet for 20 weeks had an approximately 9x higher fat content than livers of control animals receiving a normal diet. Treatment with a compound (comp) led to significant reduction of liver fat as assessed by MRI, despite continuation of the NASH diet. Biochemical analyses at the end of the study confirmed reduced lipids and triglycerides in NASH animals treated with comp. Of note, despite reducing liver fat, comp had no impact on body weight, pointing to the importance of having a non-invasive readout for liver fat. **(B)** Liver stiffness assessed by shear wave elastography (SWE). Mice receiving CCl_4_ (0.75 μL/g intraperitoneal injection 3x per week) developed significantly higher liver stiffness compared to control animals, which was consistent with biochemical and histological analyses demonstrating higher collagen content and picrosirius red (PSR) staining in the livers of CCl_4_-challenged animals. See Gapp et al. (2019) for more details. ^©^ 2019 The Authors.

Imaging techniques have also been applied to animal models (Figure 7). Mice exposed to a high fat/NASH diet had elevated serum AST and ALT levels accompanied by increased liver fat quantified by MRI (Gapp et al., 2019). Imaging demonstrated that the total liver fat progressively increased during the first 8 weeks of high fat/NASH feeding and subsequently plateaued. The MRI data were consistent with biochemical analyses of liver lipids, which were elevated at all measured time points in NASH mice, and with histology demonstrating the presence of microvesicular and macrovesicular steatosis (Gapp et al., 2019). The therapeutic efficacy of GS-0976, an acetyl-coenzyme A carboxylase inhibitor, and LJP305, a close analogue of the farnesoid X receptor agonist tropifexor, was then studied in the model. Following a high fat/NASH diet for 20 weeks, mice were treated with either GS-0976 or LJP305 while continuing to be fed the high fat/NASH diet. LJP305 and GS-0976 treatments reversed fatty liver by markedly decreasing liver fat content, as measured by MRI. However, a rebound in liver fat was observed during the last 4 weeks of treatment with GS-0976. Both drugs significantly resolved microvesicular steatosis, but macrovesicular steatosis was solely improved by LJP305 (Gapp et al., 2019). Of note, macrovesicular steatosis is primarily related to liver fat in NAFLD and microvesicular steatosis has been connected with more advanced fibrosis (Tandra et al., 2011).

Fibrosis grade was significantly related to shear-wave velocity in rats receiving CCl_4_ (Sugimoto et al., 2018). Texture analysis of SWE images further improved the diagnostic accuracy for severe fibrosis (Gu et al., 2021). In a CCl_4_ mouse model, magnetic resonance elastography and atomic force microscopy demonstrated a heterogeneous distribution of liver stiffness at macroscopic and microscopic levels, respectively, with high stiffness being attributed to areas of dense extracellular matrix (Kostallari et al., 2022). In another study, ultrasound measurements of echogenicity and stiffness in the liver were strongly correlated with macrovesicular steatosis and fibrosis, respectively, for mice submitted to a choline-deficient, L-amino acid-defined, high-fat diet to induce NASH features (Czernuszewicz et al., 2022). Also, SWE demonstrated the predisposition of mice with hepatic epithelial cell-specific deletion of leucine-rich repeat-containing G-protein–coupled receptors 4 and 5 (Lgr4/5dLKO) to liver fibrosis. These and additional data supported the concept that mice with decreased Wnt/β-catenin signaling on Lgr4/5dLKO deletion were susceptible to develop a NASH-like phenotype including fibrosis (Saponara et al., 2023).

From the 3R’s perspective, there are multiple advantages of incorporating imaging into preclinical NASH studies: enhancement of the translational power of activities by applying the same imaging techniques preclinically in the animal models and clinically in patients; the non-invasive nature of imaging supports longitudinal studies by allowing repeated assessments in the same animal, thereby contributing to the reduction (>80%) of animal usage in the experiments; imaging strongly supports therapeutic studies, by allowing randomization of animals before treatment begin.

### Safety

Besides efficacy, *in vivo* toxicology may largely profit from imaging (Reid, 2006; Wang and Yan, 2008; Hockings and Powell, 2013; Hockings and Beckmann, 2022). Indeed, non-invasive small rodent imaging is in line with the concept and strategy of toxicity testing in the 21st century developed by the National Academy of Sciences in the United States (Krewski et al., 2010). A few examples on the use of imaging for assessment of safety, a fundamental aspect of pharmaceutical research, are discussed next.

#### Brain microbleeds in Alzheimer’s disease

The deposition of amyloid-ß (Aß) as plaques in brain parenchyma and in vessels, this known as cerebral amyloid angiopathy (CAA), is an important pathological feature of Alzheimer’s disease. Lowering amyloid has thus been a therapeutic strategy in the past decades. Unfortunately, microbleeds comprising spots of attenuated signal due to the presence of hemosiderin (ferric iron, Fe^+3^) deposits were detected by MRI in the brain of Alzheimer’s patients receiving bapineuzumab, a monoclonal antibody against Aβ (Sperling et al., 2012). It was hypothesized that such microbleeds could result from altered vascular permeability due to mobilization of parenchymal or vascular Aβ (Sperling et al., 2011). The term amyloid-related imaging abnormalities (ARIA) describes MRI findings including sulcal effusion and parenchymal edema (ARIA-E) as well as hemosiderin deposition (ARIA-H), the latter referring specifically to hypointense spots on gradient-echo images (Sperling et al., 2011). In view of safety, ARIA-H has become an integral part of animal (Beckmann et al., 2011; Marinescu et al., 2017; Neumann et al., 2018) and clinical studies (Sperling et al., 2012; Arrighi et al., 2016; Lowe et al., 2021) of compounds aiming to lower Aβ deposition in the brain. Assessment of cerebral microbleeds has even been introduced as biomarker for assessing therapy effect (Chiu et al., 2020).

**FIGURE 8:**
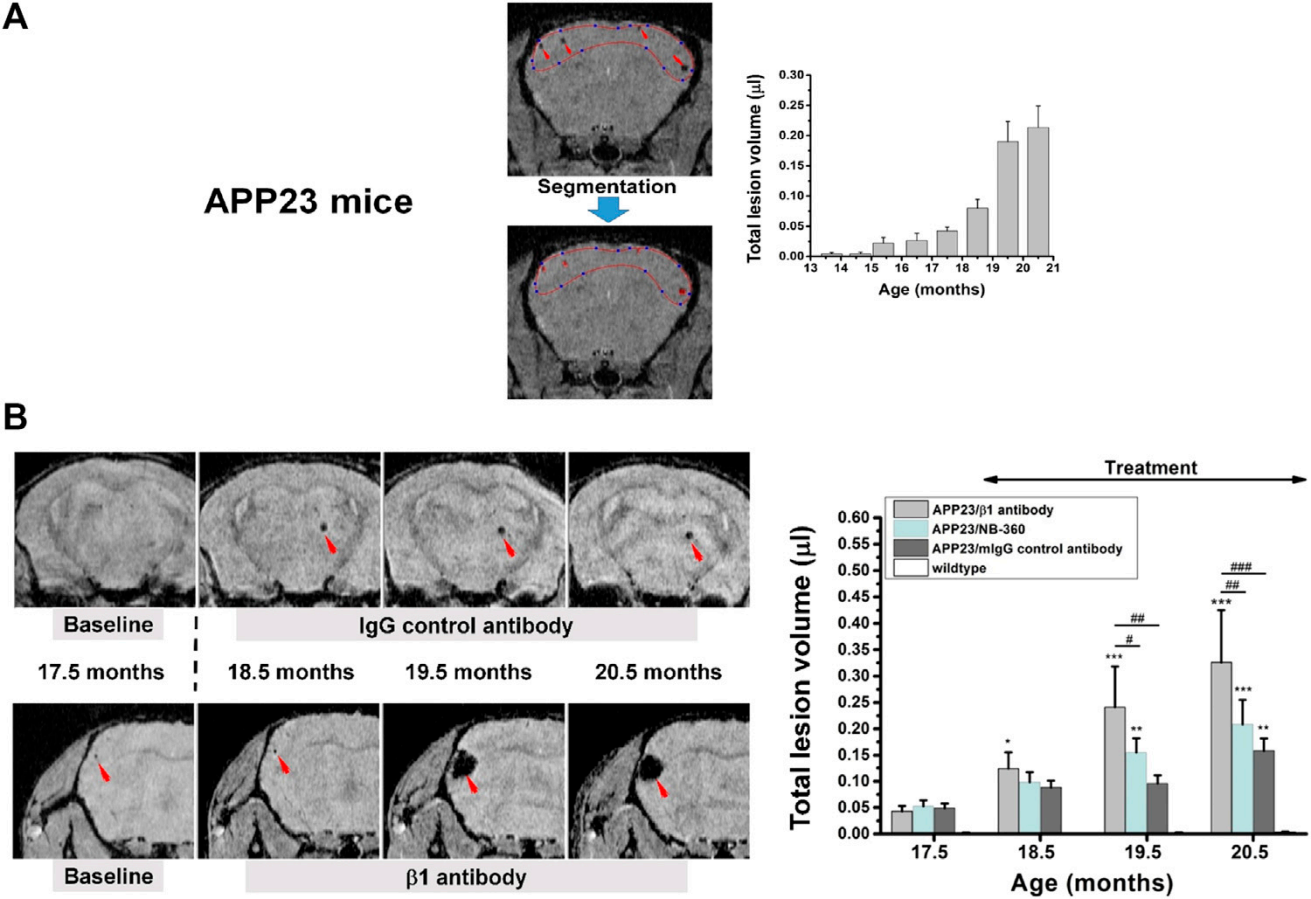
Microbleeds in the brain of APP23 mice modeling Alzheimer’s disease. **(A)** Gradient-echo MRI was sensitive to detect microbleeds from an age of 15.5 months onward. Lesion volume was quantified by segmenting the regions displaying signal attenuation. **(B)** Longitudinal images from two APP23 mice at approximately the same anatomical location for each animal. Treatment started immediately after the acquisition of baseline images at 17.5 months of age. The lesion volume over the three-month-period of treatment is summarized on the right. More details can be found in Beckmann et al. (2016). ^©^ 2016 The Authors.

In a back-translational effort, MRI was included in preclinical safety studies for the BACE-inhibitor NB-360 in APP23 mice (Beckmann et al., 2016), which in addition to parenchymal amyloid plaques also develop CAA (Winkler et al., 2001). Prior to the safety studies, the ability of gradient-echo MRI to quantify microbleeds in these mice was verified in a longitudinal characterization. Indeed, microbleeds started to develop at 15 months of age and increased significantly at later ages (Figure 8A). Based on this initial assessment, safety analyses were performed in a consecutive 3-month study on APP23 mice starting at 17.5 months of age. For the safety study, mice at age 17.5 months were examined at baseline by gradient-echo MRI, randomized in groups for microbleed volume, and then treatment followed for 3 months with a ß1-antibody known to exacerbate microbleeds (Beckmann et al., 2011), a control antibody, and NB-360. The microbleed load in the brains of APP23 mice receiving the ß1 antibody increased significantly, while APP23 mice receiving the control antibody or the BACE inhibitor displayed a microbleed development similar to that of untreated APP23 animals (Figure 8B). These data pointed to the safety of NB-360 in the model and supported the transition of the BACE-inhibitor to clinical studies (Neumann et al., 2018).

#### Kidney function in a renal safety assessment

Nucleos(t)ide analogues administered orally and primarily excreted through the kidney are commonly used for the treatment of hepatitis B and HIV (Fontana, 2009). Unfortunately, renal toxicity has been reported for some of such antiretroviral drugs (Izzedine et al., 2005; Fontana, 2009). The next example addresses a comparative renal safety assessment of four hepatitis B compounds in healthy rats: Adefovir, Tenofovir, Telbivudine, and Entecavir (Uteng et al., 2017). To this end, a functional assessment was performed with MRI: images from the kidney were sequentially acquired with a temporal resolution of 3 s/image. Following the acquisition of a number of baseline images, a contrast agent named Dotarem (Gd-DOTA) was injected intravenously during 1 s as a bolus. Dotarem, which is clinically approved, is cleared through the kidney. After the bolus injection, contrast changes due to Dotarem occurred first in the cortex and then in the medulla. Such signal profiles were then be used to estimate the time of arrival (or time-to-peak, TTP) and the amount of Dotarem in each kidney compartment. Rats were treated during 28 days with a low dose (10x the human equivalent dose) or a high dose (ranging between 25x and 40x the human equivalent dose, depending on the compound). For Adefovir, the temporal profiles of the contrast agent for the renal cortex changed considerably during the course of the study–at day 28, a significantly increased TTP as well as Dotarem amount was detected in the cortex for the higher dose of Adefovir, suggesting an impaired kidney ability to clear the agent. In other words, a toxic effect of Adefovir. Pathology examination at the end of the study revealed morphological kidney alterations affecting mainly the proximal tubules, consistent with the functional deficits detected by MRI in the renal cortex. Neither functional alterations nor significant pathological changes were detected for the other three compounds (Uteng et al., 2017).

Interestingly, nephrotoxicity was reported for Adefovir in post-marketing studies (Izzedine et al., 2005; Fontana, 2009). Overall, in this functional study MRI led not only to a reduction of animals but had also the sensitivity to quantify functional alterations that remained undetectable by standard glomerular filtration rate assessments (Uteng et al., 2017). A similar approach has also been used to study adriamycin-induced nephropathy in rats (Egger et al., 2015b).

#### Vascular leakage in the lung

A balanced signaling at the level of S1P receptors on the endothelium contributes to the functioning of vascular barriers (Marsolais and Rosen, 2009). Tight junctions between cells are stabilized by tonic S1P1 signaling, which activates endothelial nitric oxide (NO) synthase resulting in NO production and activation of the soluble guanylate cyclase, thus contributing to maintain the patency of vessels (Wilkerson et al., 2012). On the other hand, a disruption of adherent junctions and an increase in paracellular permeability are associated to S1P2 and/or S1P3 signaling (McVerry and Garcia, 2005).

Using the potent and selective S1P1 antagonist, NIBR-0213, Bigaud et al. (2016) examined potential acute and long term impact of S1P1 competitive antagonism on vascular barriers. NIBR-0213 had previously demonstrated good oral efficacy and tolerability in a mouse model of autoimmune disease but also a leakage effect in the lung (Quancard et al., 2012). Rats were treated orally during 4 days with the compound and examined repeatedly by MRI at baseline and at different time points during the course of the treatment. At 6 h, fluid signals were detected in the lungs and the pleura (Bigaud et al., 2016). Fluid volumes were reduced by 30%–40% at 24 h and could no longer be detected after 72 h, despite continuation of NIBR-0213 treatment. In other words, longitudinal MRI demonstrated an acute and transient fluid leakage induced by NIBR-0213 in the rat lungs (Bigaud et al., 2016). From the 3R perspective, inclusion of imaging as readout enabled to generate the data with 5x less animals in comparison to terminal assessments based on, e.g., Evans blue dye leakage.

## Final remarks

Besides significant reduction in animal numbers, the main asset of *in vivo* imaging within of preclinical pharmacological research lies in its ability to support translational research: applying the same technique in animal models and in the clinic enhances the confidence about the usefulness of a therapeutic agent. Moreover, back-translating learnings from the clinic contributes to refining the animal models and to enhancing the relevance of preclinical studies, since these can be guided by clinical requirements and questions as illustrated here through several examples discussed. Assessment of pharmacodynamic effects of compounds on established pathology with stratification of animals into groups based on imaging measures occurring just before treatment initiation becomes feasible.

There are important points to take into account when adopting anatomy-based imaging techniques like MRI and SWE in small rodent models: 1) The non-specific nature of the readouts. This means that, for every new application, a validation of the assessed parameters is necessary in order to verify their appropriateness and to make the link to histological or molecular analyses. In the context of pharmacology, the validation ideally comprises two steps, namely, the biological validation in which the ability of imaging to detect pathological changes is verified, followed by pharmacological validation involving testing a reference compound known to work in the model in order to analyze the sensitivity of the readout to map compound effects. Only after such extensive validation can imaging be utilized in routine pharmacological testing. In the absence of a reference compound, things become more complicated, on the other hand there is the opportunity to truly test a new therapy in an application for which earlier attempts failed; 2) the macroscopic character of the *in vivo* imaging techniques. Despite providing good spatial resolution in reasonable measurement times consistent with animal welfare, MRI and SWE are far from the microscopic world of histology, and comparisons between images obtained at different scales are in many cases challenging. SWE has in addition limited depth of penetration into the body; 3) absolute quantification of MRI parameters often comprises extensive calibration and comparison to measurements on phantoms. For instance, Na^23^-MRI may provide a measure of sodium concentration in tissue, but a complex calibration of radiofrequency inhomogeneity with a procedure not well established beyond research is necessary (Lommen et al., 2016) and the limited sensitivity make it very demanding for use in small rodents. Arterial spin labeling (ASL) enabling the quantification of tissue perfusion without administration of a contrast agent is very useful for research and clinical studies, particularly in those involving multiple longitudinal measurements. However, ASL has been confronted with a number of challenges, including relatively low signal-to-noise ratio and temporal resolution, as well as a large number of sequence variants (Jezzard et al., 2018), hampering its uptake by clinical and preclinical practice. Despite the challenges with absolute quantification, in preclinical routine relative numbers, involving for instance comparisons between assessments performed pre- and post-compound dosing, are largely sufficient in the vast majority of applications.

By law experimenters involved in *in vivo* research are required to abide to rules that take full account of the 3Rs principles. Protocols regulating experimental procedures are constantly revised and reviewed by authorities. The examples discussed previously clearly illustrate that the main benefit of *in vivo* imaging for the 3Rs lies in reducing the animal numbers followed by the potential to refine experimental procedures. Despite the fact that the replacement of animals is a constant consideration throughout the design and conduct of research programs, it needs to be shown how imaging can contribute to it. Promising alternative methods comprise organoids (*in vitro* growing human cells that form a 3D structure, allowing to study their interactions), organs-on-a-chip (human 3D microfluidic cell culture integrated chips simulating, e.g., mechanical and physiological responses of an organ or organ system) or *in silico* models (computer models without living tissue of, e.g., physiological processes) (Zhou et al., 2021; Zietek et al., 2021; Kreutzer et al., 2022; Nolan et al., 2023). Confocal, super-resolution confocal, multiphoton or light-sheet microscopy can be used for high-resolution 3D examination of cleared organoids containing fluorescence reporters and after immunolabeling (Dekkers et al., 2019). However, the complexity of human physiology involving innumerous interactions between countless, often unknown and insufficiently understood molecules or cell types remains an obstacle for a widespread replacement of animal studies by such alternative techniques at the present stage. Nevertheless, the possibility to employ learnings and data obtained *in vivo* to optimize organs-on-a-chip or *in silico* models might be a small but constructive contribution of imaging to replacement. A dialogue between *in vitro* and *in vivo* researchers in view of 3Rs is thus very important.

The ultimate goal of pharmacological research is to test new therapies in humans. Animals allow not only to gain knowledge on basic mechanisms but ensure that tests in humans are as safe as possible. Preclinical research intends to reduce the experimentation in humans, by selecting the safest and potentially most efficacious compounds to enter clinical testing. Since imaging is also an integral part of clinical compound development, imaging biomarkers/readouts may be investigated in small rodents and validated through histology before adopting them in humans. Overall, it remains the responsibility of the investigators to conceive the experiments in a way that the 3Rs principles are respected in every biomedical activity involving the use of *in vivo* imaging—a reduction of unnecessary procedures in both animals and humans in the framework of pharmacological research and development needs always to be kept in mind.

## Figures and Tables

**Table:** 

Aß	Amyloid-ß
Alk	Anaplastic lymphoma kinase
ALT	Alanine aminotransferase
ARIA	Amyloid-related imaging abnormalities
ASL	Arterial spin labeling
AST	Aspartate aminotransferase
BDNF	Brain derived neurotrophic factor
CAA	Cerebral amyloid angiopathy
CCl_4_	Carbon tetrachloride
CMT	Charcot-Marie-Tooth
COV	Coefficient of variation
CRISPR	Clustered regularly interspaced short palindromic repeats
CT	Computed tomography
DTI	Diffusion tensor imaging
EAE	Experimental autoimmune encephalomyelitis
ECM	Extracellular matrix
fMRI	Functional MRI
JTA	Janus Tough Adhesive
LPS	Lipopolysaccharide
MRI	Magnetic resonance imaging
MRI-PDFF	MRI-derived proton density fat fraction
MS	Multiple sclerosis
MSU	Monosodium urate
MTR	Magnetization transfer ratio
NO	Nitric oxide
NSCLC	Non-small cell lung cancer
OA	Osteoarthritis
OARSI	Osteoarthritis Research Society International
phMRI	Pharmacological MRI
PMP22	Peripheral myelin protein-22
rs-fMRI	Resting state functional MRI
S1P	Sphingosine-1-phosphate
SD	Standard deviation
SPECT	Single photon emission computed tomography
TCPOBOP	1, 4-bis [2-(3, 5-dichloropyridyloxy)] benzene
TREM2	Triggering receptor expressed on myeloid cells 2
TTP	Time-to-peak
US	United States
UTE	Ultrashort echo time
